# Enrichment of SOX2-Positive Cells in BRAF V600E Mutated and Recurrent Ameloblastoma

**DOI:** 10.3390/jpm12010077

**Published:** 2022-01-08

**Authors:** Chih-Huang Tseng, Pei-Hsuan Lu, Yi-Ping Wang, Julia Yu Fong Chang

**Affiliations:** 1 Division of Oral Pathology & Maxillofacial Radiology, Department of Dentistry, Kaohsiung Medical University Hospital, Kaohsiung 80756, Taiwan; 1050565@kmuh.org.tw; 2 Oral & Maxillofacial Imaging Center, College of Dental Medicine, Kaohsiung Medical University, Kaohsiung 80708, Taiwan; 3 Graduate Institute of Clinical Dentistry, Department of Dentistry, National Taiwan University, Taipei 100229, Taiwan; ypwang0530@ntu.edu.tw; 4 Department of Dentistry, National Taiwan University Hospital, College of Medicine, National Taiwan University, Taipei 100229, Taiwan; R04422025@ntu.edu.tw; 5 Institute of Oral Biology, School of Dentistry, National Taiwan University, Taipei 100229, Taiwan

**Keywords:** ameloblastoma, SOX2, stem cells, BRAF V600E, recurrence

## Abstract

Ameloblastoma is the most common benign odontogenic neoplasm, but with an aggressive behavior and a high recurrence rate. Nowadays wide surgical resection is the current recommended treatment, which can cause further loss of function and esthetics. Recent studies point to the stem/progenitor cells as both initiators and propagators of the tumors. Elucidation of the cellular and molecular mechanisms underlying the tumor stem cells is of broad interest for understanding tumorigenesis and for developing effective targeted therapies. SRY related HMG box gene 2 (SOX2) is a transcription factor that plays important roles in development, stem cell renewal, and cancer formation. Few studies have revealed increased SOX2 expression in atypical ameloblastoma and ameloblastic carcinoma. For the development of personalized medicine for ameloblastoma, biomarkers that provide prognostic or predictive information regarding a tumor’s nature or its response to treatment are essential. Thus, in this study, we aimed to study if SOX2-positive cells exist in ameloblastomas and their correlation with the clinicopathologic parameters. Our data suggested BRAF(V600E) mutation might contribute to the expansion of SOX2-positive cells. The identification of BRAF(V600E) mutation and the amplification of SOX2-positive cells in ameloblastomas imply the possible benefit of applying BRAF and SOX2 inhibitors in recurrent and un-resectable ameloblastomas.

## 1. Introduction

Ameloblastoma is the most common odontogenic neoplasm, but with a high recurrence rate. Ameloblastomas are divided into three subtypes by WHO classification of tumors [1], namely, solid/multicystic type (AM-S/M), extraosseous/peripheral type, and unicystic type. These subtypes differ in clinicoradiographic presentations and prognosis. The AM-S/M is the most common subtype with an aggressive clinical behavior. Although AM-S/M tumors are slow growing, they are locally invasive, which causes considerable tissue destruction and subsequent morbidity. In addition, the AM-S/M tends to infiltrate in between cancellous bone trabeculae beyond the radiographical margin, and this feature leads to high recurrence rate if adequate surgical margins are not acquired [1]. The recurrence rate of AM-S/M has been reported up to 50 to 90% after curettage, and an alarming recurrence rate of 15% even after marginal or block resection [2]. Recent advances of molecular biology unraveled that recurrent BRAF(V600E) activating mutation is the most common genetic aberration in ameloblastomas [3,4,5], however, the detailed tumorigenic molecular mechanism is yet to be fully elucidated.

Although the pathogenesis of the ameloblastoma has been pursued for years, the origin of this tumor remains unknown. The stem cells play a major role in the regulation of tissue homeostasis. The adult stem cells are long-lived and have the ability of self-renewal and multi-lineage differentiation [6]. If the proliferative capacity of stem cells becomes uncontrolled and the differentiation potential become impaired, these self-renewable stem cells tend to have the potential to cause tumor initiation [7]. Several markers are proposed for identification of tissue-specific stem cells [8,9], however, there is no universal marker for adult stem cells. In regard to odontogenesis, some dental epithelial stem cell (DESC) markers have been identified. For example, the active stem cell marker, Lgr5, has been found in the stellate-reticulum(SR) region of the cervical loop [10]. Furthermore, in vivo lineage tracing experiments further show that the SOX2-positive DESCs give rise to multiple lineages of dental epithelial cells [11]. SOX2 is a transcription factor of the SOX family. The SOX family plays various roles during development, in adult tissue homeostasis and regeneration, and in fate decisions of stem and progenitor cell and differentiation [12]. SOX2 is expressed in multiple epithelial tissues, such as oral epithelium, cervix, anus, testes, lens, and multiple glands, and the SOX2-expressing cells are demonstrated to continuously generate mature cell types in experiments of genetic lineage tracing and transplantation [13]. SOX2 is also involved in multiple tumor cell functions, such as the promotion of tumor cell proliferation [14,15,16], the ability to repress apoptosis [16,17], acceleration of cell invasion and migration [18,19,20], and regulation of self-renewal in tumor stem cell populations [19,21]. SOX2 expression also has been correlated with clinical parameters, such as staging, relapse, therapy resistance, and prognosis of patients in various cancers [20,22,23,24,25,26,27]. In addition, BRAF(V600E) mutation was shown to be associated with upregulation of SOX2 [28]. However, conflicting data regarding the expression profile of SOX2 in ameloblastoma are present in the contemporary literatures [29,30]. As a consequence, the biological significance of SOX2 in this odontogenic neoplasm and its clinical relevance are still shrouded in mystery. Thus, providing a defined detection method for SOX2 in ameloblastoma, elucidating the identity as active or quiescence stem cells, and investigating the roles in drug response will be beneficial for developing personalized medicine in the future.

In this study, we first compared two different antibodies for precise detection of SOX2 in ameloblastomas, then explored the immunohistological staining patterns of SOX2 across the variants of ameloblastoma and verified their concordance to the sequencing results of BRAF. Correlations of SOX2, Ki-67, and clinicopathological parameters were also performed. We also demonstrated that knockdown of SOX2 leads to decreased viability of ameloblastoma cells, and increased expression of SOX2 was associated with resistance to anti-BRAF small molecule inhibitors, vemurafenib and dabrafenib, in ameloblastoma cells. Our study demonstrated a good example of how to verify a biomarker from clinical aspect and examine the biological function of the molecule toward precision medicine.

## 2. Materials and Methods

### 2.1. Patients and Specimens

Seventy-four formalin-fixed, paraffin-embedded tissues of ameloblastoma were collected in this study. All tissue blocks were obtained from the Department of Oral Pathology, National Taiwan University Hospital, Taiwan, from 2007 to 2016. The diagnosis was based on histological examination of hematoxylin and eosin-stained tissue sections by four board certified oral pathologists (Chun-Pin Chiang, Bu-Yuan Liu, Julia Yu Fong Chang, and Yi-Ping Wang). All of these specimens were acquired from biopsy, curettage, excision, or enucleation without decalcification. The 74 enrolled cases were divided as follicular type (age 18 to 79 years, mean age 47.2 years), plexiform type (age 12 to 77 years, mean age 30.9 years), and unicystic type (age 11 to 66 years, mean age 29 years). The detailed epidemiologic data are summarized in Table 1. Six dental follicles of impacted third molars from 6 patients with the presence of numerous remnants of odontogenic epithelial rests were also collected as the control group of dental epithelia. This study was approved by the Research Ethics Committee of National Taiwan University Hospital (No. 201412058RINA, 201608088RINA and 201901034RIND).

### 2.2. Immunohistochemistry and Immunofluorescence of SOX2 and Ki67

Immunohistochemical stain was performed using previously described protocols [31]. The primary antibodies for SOX2 staining were rabbit anti-SOX2 IgG (1:500–1:1,000 dilution; Millipore, Billerica, MA, USA) and anti-SOX2 (1:100 dilution; 3579S; Cell Signaling, Danvers, MA, USA), and they were incubated with the sections at 4 ℃ overnight. For Ki-67 staining, the monoclonal antibody (1:100 dilution; clone MIB-1; Dako, Agilent, Santa Clara, CA, USA) was incubated for 20 min in room temperature (RT).

For double immunofluorescence assay, six cases of ameloblastoma (2 follicular, 2 plexiform, and 2 unicystic type) with high level of SOX2 and Ki-67 expression were pretreated and incubated with primary antibodies against SOX2, as described in immunohistochemical staining. After washing the slides with PBST_0.1_ three times for 5 min, incubation with secondary antibody (1:300; Alexa Fluor 546-conjugated goat anti-rabbit IgG; Thermo Fisher Scientific, Waltham, MA, USA) was completed at RT for 1 h. After washing PBST 5 min for 3 times, these slides were incubated with Ki-67 monoclonal antibody (Dako) for 20 min. After washing, slides were incubated in secondary antibody (1:300; Alexa Fluor 488-conjugated goat anti-mouse IgG; Thermo Fisher Scientific) for labeling Ki67+ cells at RT for 1 h. Then, we washed these slides in PBST for 5 min 3 times, the nuclei of tumor cells were stained with 4′,6-diamidino-2-phenylindole (DAPI) (Thermo Fisher Scientific), then washed in PBST for 5 min and mounted with cover slides by Prolong^®^ Diamond Antifade Mountant (Thermo Fisher Scientific).

### 2.3. Assessment

For assessing immunohistochemically stained sections, digitized images were captured. Ten views of every case were randomly selected under 200-fold magnification for quantification. If lesser than ten views could cover all the tumor area, we counted all tumor parts in the section. All histopathological images were taken with Olympus BH-2 microscope and DP2-BSW image acquisition software (Olympus, Tokyo, Japan). Only tumor cells with nuclear staining were regarded as positive cells. The quantification of positive tumor cells to SOX2 and Ki-67 and the numbers of total tumor cells were counted by Image J and IHC profiler Plugin (National Institutes of Health, Bethesda, MD, USA). The labeling index was calculated as the percentage of SOX2 and Ki-67-positive nuclei to all tumor nuclei.

The clinical parameters, such as bone perforation and root resorption caused by tumor were acquired from records of surgical findings, pathological reports, and radiographic examinations. The size of lesions was measured from cases with pre-surgical panoramic X-ray by utilizing ROI manager of Image J. The digitalized panoramic X-ray were available in 22 of 25 cases in follicular type, 20 in 22 cases of plexiform type, and 22 in 25 cases of unicystic type.

### 2.4. Cell Lines and Cultures

Ameloblastoma cell lines (AM1 and AM3) were generous gifts from Dr. Shosei Kishida from Kagoshima University in Japan. These cells were cultured in KSFM (Defined Keratinocyte serum free medium; Gibco, Thermo Fisher Scientific) in a humidified incubator with 5% CO_2_ at 37 °C.

### 2.5. Cell Viability Assay

To measure the viability of ameloblastoma cells, alamar blue assay was performed. The cells were cultured in 24-well plates, and then alamar blue solution (Thermo Fisher Scientific) was added to each well according to the manufacturer’s protocol. After incubation for 1–3 h at 37 °C, the culture medium containing alamar blue solution was collected for further analysis. Both absorbance and fluorescence were detected using the Dynatech microplate reader (Dynatech Medical Products, Billingshurst, West Sussex, UK) with excitation at 544 nm and emission at 590 nm. The absorbance and fluorescence were taken as proportional to the number of cells present and were expressed as a percentage of the respective positive and negative controls.

### 2.6. Knockdown of SOX2

For SOX2 knockdown, AM1 and AM3 cells were transfected by lentiviruses with short hairpin RNAs cloned into the pLKO.1 vector. A non-specific shGFP-pLKO.1 was used as negative control. The shRNA and lentiviruses reagents were purchased from the RNAi Core Facility (Academia Sinica, Taipei, Taiwan). Stable cells were selected in the presence of puromycin for 1 week.

### 2.7. Immunocytofluorescence Assay

Cells were seeded onto chamber slides and fixed with 2% paraformaldehyde for 20 min and permeabilized with 0.1% Triton X-100 for 10 min. After washing, cells were blocked with 1% bovine serum albumin (BSA) in PBS for 30 min. Cells were stained with antibody against SOX2 (1:100 Cell Signaling, Danvers, MA, USA) for 60 min at RT, followed by incubation with secondary antibody (1:300; Alexa Fluor 488-conjugated goat anti-rabbit IgG; Thermo Fisher Scientific) for 60 min at RT. Nuclei were counterstained with DAPI (Thermo Fisher Scientific). Cells were observed under Olympus BX53 fluorescence microscope (Olympus, Tokyo, Japan).

### 2.8. PCR and Sanger Sequencing

Fifty-five cases with sufficient amounts of tissue were used for macro-dissection of tumor component for DNA extraction and PCR amplified. DNA was extracted by using AllPrep DNA/RNA FFPE kit (Qiagen, Germantown, MD, USA). Sanger sequencing for BRAF(V600E) mutation was then performed. Primers used in DNA sequencing are listed in Supplementary Table S1.

### 2.9. Treatment of BRAF Inhibitors

BRAF inhibitors, vemurafenib and dabrafenib (MedChemExpress, Monmouth Junction, NJ, USA), were added in the cell culture medium using measured IC50 concentration for 72 h before detecting cell viability. The resistant clones were developed using IC50 concentration for selection and subcultured for at least 2 passages.

### 2.10. RNA Purification, Reverse Transcription, and Quantitative Real-Time PCR (qRT-PCR)

Total RNA was extracted from AM1 and drug treated AM1 cells, and reverse transcription was subsequently performed. qRT-PCR was performed using the Light Cycler^®^ 480 SYBR Green I Master kit (Roche Applied Science, Indianapolis, IN, USA) and the LightCycler480 System (Roche Applied Science). The gene expression levels of each sample were normalized to the expression levels of GAPDH. Primer sequences used were listed in Supplementary Table S1.

### 2.11. Western Blot Analysis

Western blots were performed as previously described [32]. The membrane was incubated with antibody against Anti-P Glycoprotein 1 antibody (1:1000 Abcam, Cambridge, UK), BRAF(V600E) (VE1) (1:2000 Spring Biosciences, Abcam), ERK (1:1000 Cell Signaling), phosphor-ERK (1:1000 Cell Signaling), SOX2 (1:1000 Cell Signaling), AKT (1:1000 Cell Signaling), phosphor-AKT (1:1000 Cell Signaling), α-tubulin (1:5000 Cell Signaling).

### 2.12. Statistical Analysis

Data were entered into the Statistical Package for Social Sciences (SPSS) program, Version 23 (SPSS Inc., Chicago, IL, USA). The associations between clinicopathological parameters and the expression status of SOX2 and Ki-67 were analyzed by Mann–Whitney U test. Spearman’s correlation coefficient was used to identify the correlation between SOX2 and Ki-67 expression status, and between expression of both markers and clinicopathological parameters. The difference between two experimental groups in all in vitro studies and correlation between SOX2-positive cell numbers and BRAF status in ameloblastomas were evaluated by *t*-test. A *p* value of <0.05 was considered significant. All values are represented as mean ± standard deviation.

## 3. Results

### 3.1. Verifying Specificity of Antibody for Detection of SOX2-Distinct Expression Patterns of SOX2 in Three Types of Ameloblastoma under Different Anti-SOX2 Antibodies

**Figure 1:**
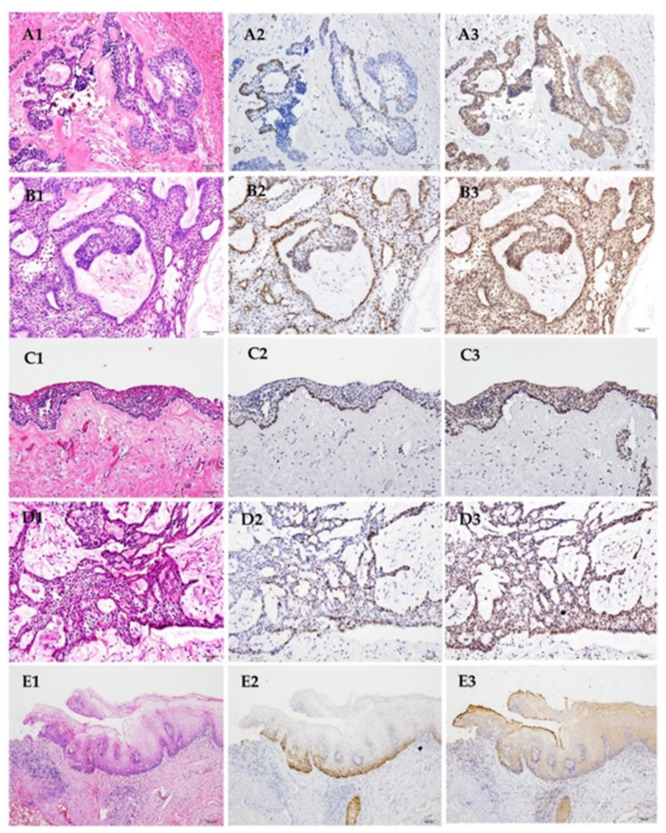
Different types of ameloblastoma with hematoxylin and eosin (H&E) stain and immunohistochemical stains (IHC). Histopathological subtypes were as followed: follicular (**A1**–**3**), plexiform (**B1**–**3**), and unicystic (**C1**–**3**,**D1–3**). The (**D**) series are the intraluminal portion of a unicystic ameloblastoma. The (**E1**–**3**) are normal oral squamous epithelium including in the specimen, serving as an internal positive control. (**A2**,**B2**,**C2**,**D2**,**E2**: 1:100 dilution (Cell Signaling); **A3**,**C3**,**D3**,**E3**: 1:1000 dilution (Millipore); **B3**: 1:500 dilution (Millipore). Original magnification ×200 in **A**–**D**; ×100 in **E**).

The conflicting results regarding the SOX2 expression pattern in ameloblastomas are most likely due to using different antibodies. As the first step toward precision medicine, a defined and precise detection method for SOX2 is required. Thus, we first examined the expression patterns of SOX2 using two different antibodies, which have been used in the literature [29,30], in 15 ameloblastoma cases. All fifteen cases were positive for both anti-SOX2 antibodies. However, the expression patterns of the two primary antibodies were notably different (Figure 1). The positive signal of 3579S from Cell Signaling was scattered and was detected at the nuclei of the tumor cells. The signal was predominantly seen in the peripheral ameloblast-like cells and focally positive in SR-like cells. (A2, B2, C2, D2). The intensity of stain was variable between areas and cases. On the other hand, anti-SOX2 antibody of Millipore resulted in a diffuse nuclear and focal cytoplasmic stain in both ameloblast-like cells and SR-like cells in all cases (A3, B3, C3, D3). The surrounding stromal cells were mostly SOX2-negative. In normal oral squamous epithelium, the positive signals of SOX2 were restricted at the basal and parabasal layers when incubated with 3579S of Cell Signaling (Figure 1E2). However, a diffuse non-specific stain of SOX2 was discerned in keratinocytes in the full thickness of the surface epithelium when interrogated with the Millipore antibody (Figure 1E3). According to the expression pattern, we chose 3579S of Cell Signaling for our subsequent assays of SOX2 in a larger cohort study, due to better specificity.

### 3.2. Remnants of Odontogenic Epithelium in Dental Follicle Containing SOX2+ Cells

**Figure 2:**
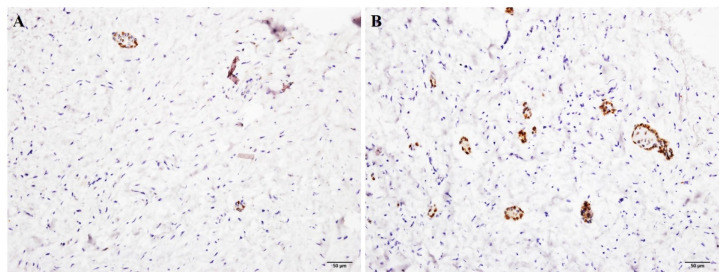
Remnants of odontogenic epithelium in dental follicle containing SOX2+ cells. (Immunohistochemical staining showing SOX2+ cells in dental follicular tissue with (**A**) few remnants and (**B**) more remnants.) (Original magnification ×200).

All six cases of odontogenic epithelial rests in dental follicles showed positive SOX2 stain in a scattered pattern. Most positive cells were located in the periphery of the nests (Figure 2) and the detail labeling indices were shown in Table 2. Thus, it is important to know that SOX2 positivity does not imply neoplasm.

### 3.3. Expression of SOX2 in Ameloblastoma

**Figure 3:**
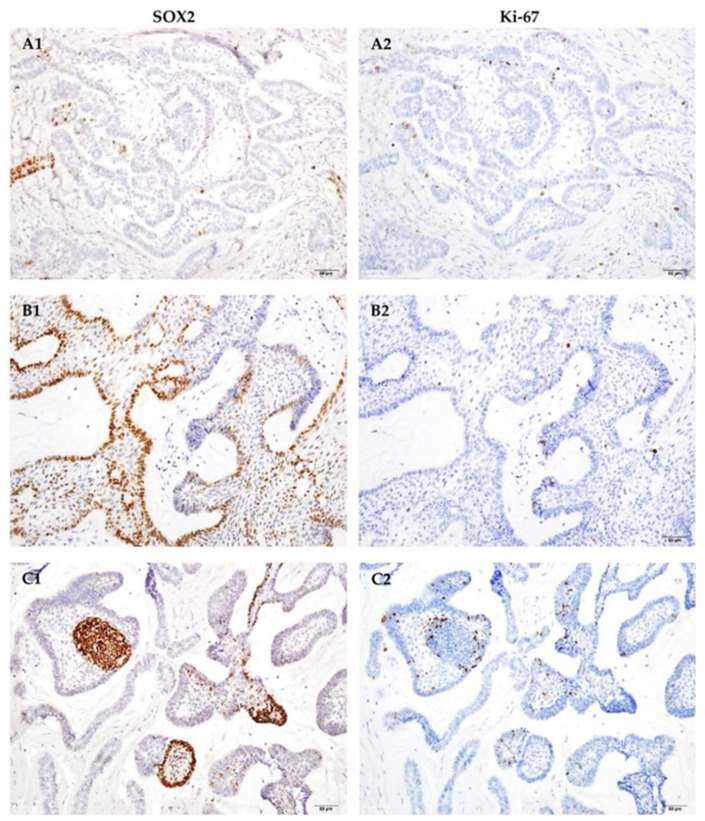
Comparison of the location of positive cells under SOX2 and Ki-67 immunostain. The cells show exclusively positive to one of these two antibodies. (**A**) follicular type; (**B**) plexiform type; (**C**) unicystic type, mural subtype. (Original magnification ×200).

The expression patterns of SOX2 in ameloblastoma to anti-SOX2 antibody crosses the histological subtypes. It showed scattered nucleus stain in the majority of peripheral pre-ameloblast/ameloblast-like cells and focal SR-like cells in follicular type AMs (Figure 1A2 and Figure 3A1) and plexiform AMs (Figure 1B2 and Figure 3B1). In the unicystic group, most positive cells were also seen in the basal cell layer reminiscent of ameloblast-like cells (Figure 1C2). The expression pattern of SOX2 in the intra-luminal and mural extension of unicystic AM was identical to that seen in the follicular and plexiform AMs (Figure 1D2 and Figure 3C1). Labeling indices for SOX2 immunostaining of these 74 cases were present in Table 3 and Table 4. The tumors of plexiform type showed the highest mean labeling index of SOX2 immunostaining, followed by unicystic and follicular type (Table 4).

### 3.4. Expression of Ki-67 in Ameloblastoma

All cases except one unicystic AM were positive to Ki-67 immunostain. Most of the positive cells were located in the second and first layer of the peripheral ameloblast-like cells, and some in central SR-like cells (Figure 3(A2,B2,C2)). Labeling indices for Ki-67 immunostaining of our tested cases were present in Table 3 and Table 5. The plexiform type AM showed the highest labeling index to Ki-67 immunohistochemical stain, and it was followed by unicystic type and follicular type AMs (Table 5).

### 3.5. Cells Expressing SOX2+ or Ki-67+ Are Located in Different Populations, Suggesting SOX2+ Cells Are Most Likely Quiescent Stem Cells

**Figure 4:**
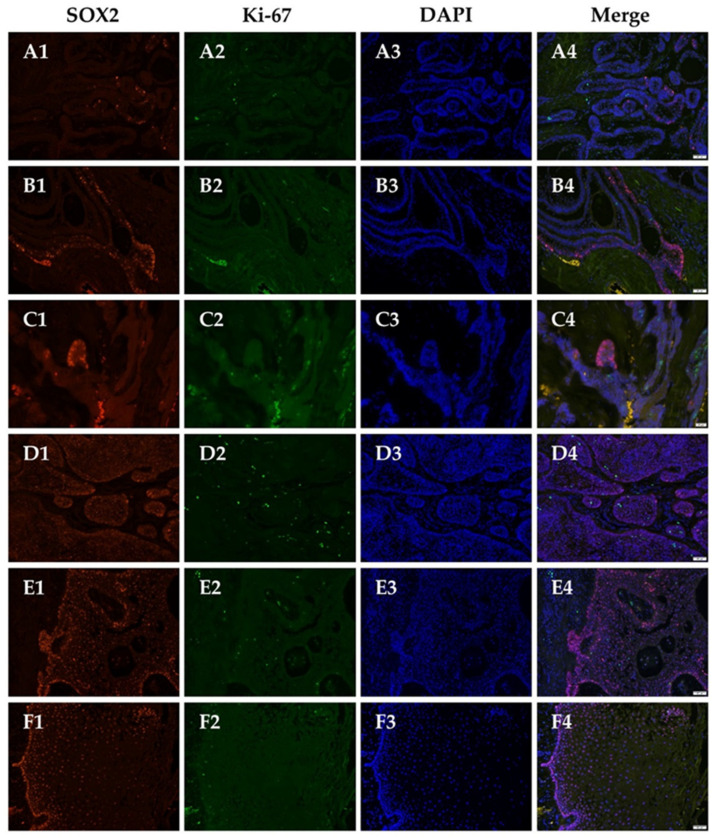
Immunofluorescence for SOX2 and Ki-67. Confirming SOX2+ and Ki-67+ cells were in different population of tumor cells. (**A**) Follicular type; (**B**,**C**) plexiform type; (**D**) follicular type with soft tissue invasion; (**E**) plexiform type with heavy inflammation; (**F**) oral squamous epithelium. (Original magnification ×200; C series: ×400).

When reviewing these immunostaining slides, we noted the cells which showed positive to anti-SOX2 antibody were negative to Ki-67, and vice versa (Figure 3). However, no correlation between the expression status of these two markers was noted (Supplementary Table S2). For further verification, we performed immunofluorescence in six cases of AM showing high expression level of SOX2 and Ki-67 to validate the spatial correlation of these two markers. Under immunofluorescence, we found that most of the SOX2-positive cells were located in the periphery of the tumor island with some in the central area, and the Ki-67+ cells located in the first and second layer of the peripheral cells (Figure 4A–C series). Furthermore, these two markers were generally mutually exclusive on the cellular level. Scant double-labeling cells were identified in three cases. One case was a follicular type with soft tissue invasion, which contained minimal inflammatory cells infiltration in the stroma (Figure 4D series). The other two cases were plexiform type, which showed dense infiltration of inflammatory cells in the fibrous stroma with proliferation of tumor epithelial cells (Figure 4E series). Squamous epithelium included in the specimen of ameloblastoma was utilized as a positive control (Figure 4F series). SOX2+ cells were involved from basal to spinous layer of the epithelium, but the intensity was greater in the cells of basal and parabasal cell layers, and Ki-67+ cells located in parabasal layer.

### 3.6. Correlation of Expression Status of SOX2 and Ki-67 with Clinicopathological Parameters and Findings

#### 3.6.1. Clinical Parameters—Recurrent Lesions Showed Higher SOX2 Positivity Comparing to Original Samples

Statistically, no significant correlation between the labeling indices of these two markers and lesional size was identified. In addition, the labeling indices of SOX2 and Ki-67 failed to correlate with bone perforation and root resorption caused by the tumor (Supplementary Table S3). Interestingly, no correlation between the size of lesions with the presence of root resorption and bone perforation is identified in three types of ameloblastoma (Supplementary Table S4).

**Figure 5:**
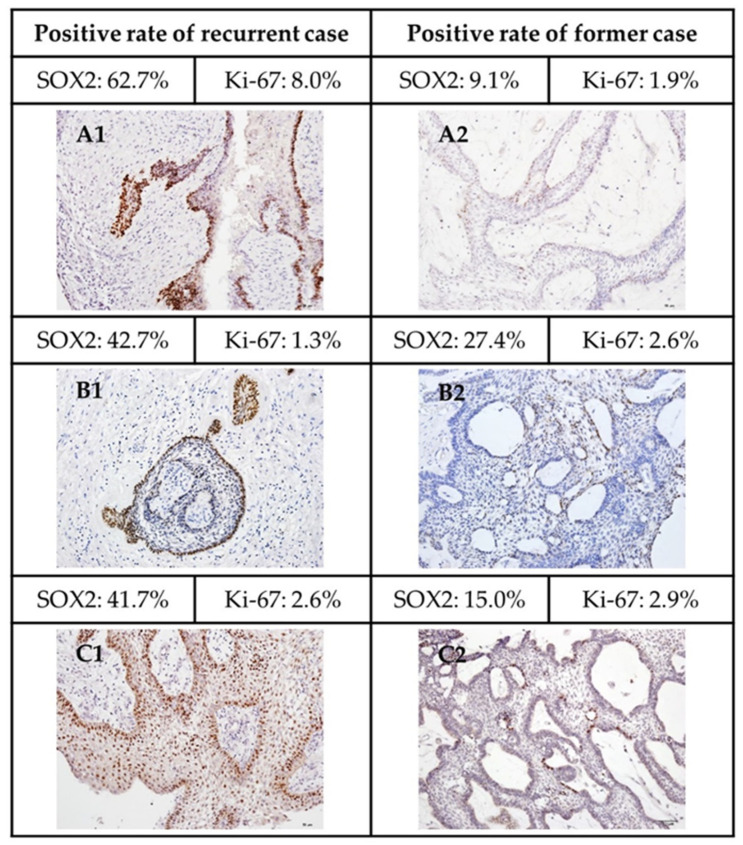
SOX2 and Ki-67 IHC labeling index in three paired cases of primary and recurrent lesions in three patients. The representative photographs were taken from three patients’ primary and recurrent lesions. (**A1**: recurrent and **A2**: primary lesions of patient 1; **B1**: recurrent and **B2**: primary lesions of patient 2; **C1**: recurrent and **C2**: primary lesions of patient 3).

Among 74 enrolled cases, 13 cases (9 cases of follicular, 3 cases of plexiform, and 1 case of unicystic type) were obtained from recurrent lesions (Supplementary Table S5), and 7 other cases (3 are in follicular group and 4 are in plexiform group) developed recurrent lesion later (Supplementary Table S6). No significant difference in both labeling indices is seen between those recurrent cases and primary lesions of the same histological subtypes. Three paired primary and recurrent lesion cases from the same patients were obtained (Figure 5A–C). The recurrent cases showed much higher labeling indices of SOX2 than paired primary lesions, however, the difference of labeling indices of Ki-67 remains to be inconclusive.

#### 3.6.2. Pathological Parameters and Findings

To see if the labeling indices of SOX2 and Ki-67 can serve as a biomarker for the presence of mural extension of unicystic ameloblastoma, we interrogated these indices between mural type and intra-luminal/luminal type unicystic ameloblastoma. No significant difference in SOX2 and Ki-67 labeling indices was noted between these two types (Supplementary Table S7). In addition, no significant difference was noted between the intra-luminal/luminal unicystic ameloblastoma and the combined group of mural type unicystic ameloblastoma and conventional solid ameloblastoma of follicular and plexiform types (Supplementary Table S8).

### 3.7. SOX2 Knockdown in Ameloblastoma Cell Lines Reduced Cellular Viability

**Figure 6:**
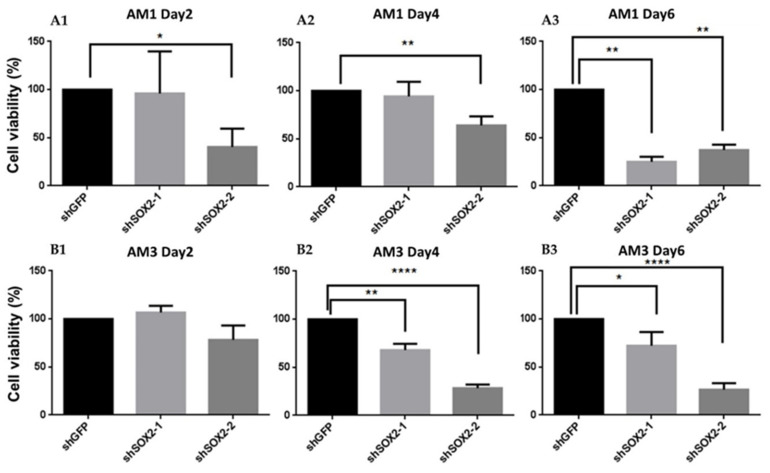
SOX2 knockdown in ameloblastoma cell lines reduces the cell viability. (**A1–3**,**B1–3**) The cell viability of AM1 and AM3 cells at 2 days, 4 days, and 6 days post SOX2 suppression. * *p* < 0.05; ** *p* < 0.01; **** *p* < 0.0001.

In order to investigate the impact of SOX2 on the viability of ameloblastoma, we silenced SOX2 by shRNA through lentiviral system in ameloblastoma cell lines (AM1 and AM3). The knockdown efficiency was checked with fluorescence observation of GFP (as a reporter) and Western blot, and confirmed valid inhibition of SOX2 (Supplementary Figure S1). Knockdown of SOX2 prominently downregulated the cell viability of both ameloblastoma cell lines (Figure 6).

### 3.8. Increased SOX2-Positive Cells in BRAF(V600E) Mutated Ameloblastomas

**Figure 7:**
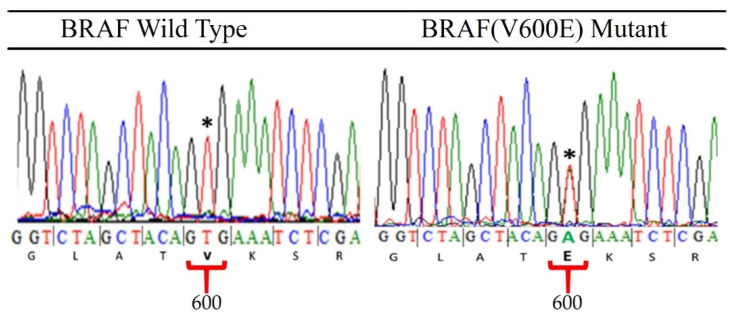
Representative wild type BRAF and BRAF(V600E) mutant sequence chromatograms of BRAF exon 15 (portion) were shown. * The BRAF c.1799 T > A mutation resulting in a heterogeneous thymine-to-adenine transversion at nucleotide position 1799, which results in a valine-to-glutamate substitution at residue 600 (BRAF V600E mutation rs113488022) was confirmed to be present in ameloblastomas.

Fifty-five ameloblastoma cases, including 18 cases of follicular type, 16 cases of plexiform type, and 21 cases of unicystic type were sent for Sanger sequencing. Forty-eight cases harbored BRAF(V600E) mutation (Figure 7).

**Figure 8:**
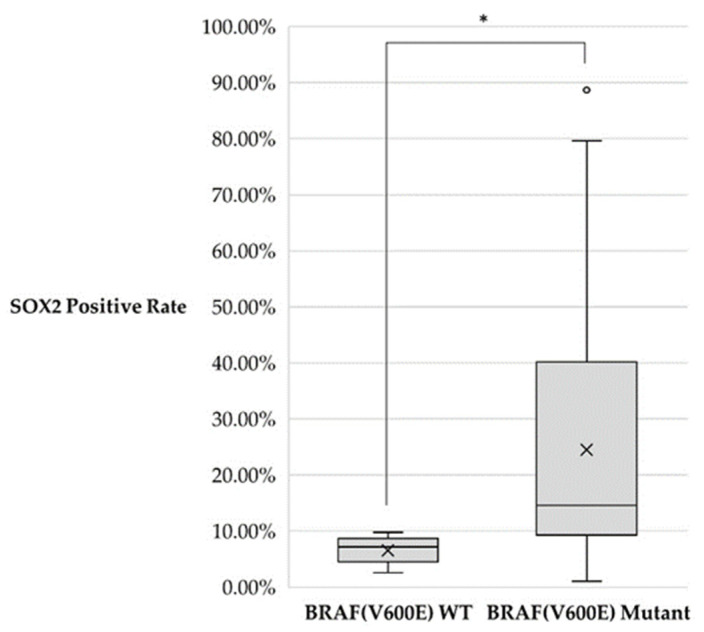
SOX2-positive rate distribution among BRAF(V600E) wild type (WT) and mutant cases. * *p* < 0.05.

SOX2-positive cells were found in all cases regardless of BRAF status, with an average of 22.5% SOX2-positive cells in ameloblastomas. BRAF(V600E)-mutated ameloblastoma cases showed significantly more SOX2-positive cells (24.5%) than in wild type (6.6%) (*p* < 0.05) (Figure 8 and Table 6).

### 3.9. Ameloblastoma Resistant Clones Show Upregulation of SOX2 Expression

**Figure 9:**
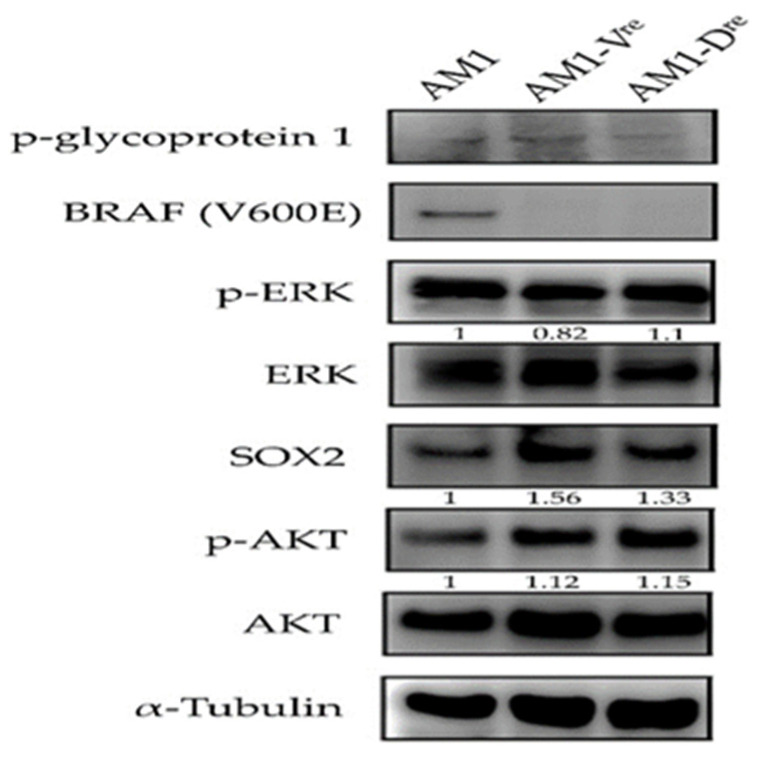
Western blot analysis of AM1 and its resistant clones to BRAF inhibitors. The AM1 cells express BRAF(V600E), while it was downregulated in the treated groups. BRAF inhibition induces upregulation of stemness marker SOX2 in AM1.

The ameloblastoma cell line AM1 had been shown harboring BRAF(V600E) mutation in previous studies [4,5], which was also confirmed in our investigation (Figure 9). The cell viability of AM1 cells were reduced by treatment of BRAF inhibitors vemurafenib and dabrafenib (Supplementary Figure S2). Then, we developed the resistant clones of AM1 cells after a long-term treatment with individual inhibitor. The AM1 vemurafenib resistant clone (AM1-Vre) and AM1 dabrafenib resistant clone (AM1-Dre) revealed significantly downregulated BRAF(V600E) and upregulated SOX2 mRNA levels compared to untreated cells. In the Western blot analysis, the expression of SOX2 was found to be upregulated in the resistant clones (Figure 9).

## 4. Discussion

Ameloblastoma is a tumor from odontogenic epithelial origin. It is thought to arise from rests of dental lamina, from a developing enamel organ, from the epithelial rests of Malassez cells, from lining of an odontogenic cyst in intrabony lesions, and from the basal cell layer of oral mucosa in peripheral type. Among these candidate tissue origins, the rests of dental lamina are the most promising origin of ameloblastoma. After the process of tooth development is complete, the dental lamina will degenerate to remnants of odontogenic epithelium retained in the jaws. For observing the expression pattern of SOX2 in dental lamina cells, it is ideal to perform the SOX2 immunohistochemistry on a developing tooth bud. However, the sections of developing tooth buds are difficult to obtain. Therefore, we compromised using dental follicle which contained remnants of odontogenic epithelium as our resource of dental epithelium.

SOX2 is a stem cell marker, and the SOX2+ dental epithelial stem cells were shown to give rise to all lineages of dental epithelial cells [11]. As these SOX2+ cells have the capacity to form the epithelial component of a new tooth, SOX2+ cells in dental lamina were thought to be dental epithelial stem cells. Moreover, SOX2+ cells were located by immunohistochemistry in dental lamina of human developing tooth germ [29]. Since the most accepted origin of ameloblastoma was the dental lamina cells, we also found that cells in epithelial rests of dental follicle showed SOX2 expression. These SOX2+ dental lamina cells might play a significant role in the pathogenesis of ameloblastoma.

The SOX2 expression pattern of ameloblastomas was scattered with variable intensity as the expression pattern we observed in odontogenic epithelial rests. Among these three histological subtypes of ameloblastoma, the highest percentage of the tumor stained with SOX2 was the plexiform type. Most of the positive cells were located in the peripheral cells with lesser in the SR-like cells. Plexiform type ameloblastoma consists of anastomosing cord-like odontogenic epithelium and is supported by matured fibrous tissue. The epithelial components are comprised of peripheral ameloblast-like cells with lesser or absent presentation of SR-like cells. The histological features should be the reason for why the plexiform type had the highest labeling index of SOX2 immunostain. We also noticed that the cells with different morphology might have a distinct expression to SOX2. The majority of the positive cells were round or oval in shape, as the features of primitive cells, but most of the peripheral pre-ameloblast ⁄ ameloblast-like high columnar cells with reverse polarity were negative to SOX2 [33]. These different appearances of tumor cells resemble the morphologic change in the process of amelogenesis. In the process of amelogenesis, the precursor cells of ameloblasts are cuboidal or low columnar in shape, then they convert to long columnar cells as ameloblasts in the later stage [11,34]. Since SOX2 has been considered as a stem cell marker [35], it is expressed in premature cells or cells with stem cell properties. This finding implied the long columnar polarized ameloblasts are differentiated cells, while these round or oval cells are undifferentiated, premature cells. Even though the tumor cells within ameloblastoma are not true ameloblasts, we propose the morphological difference of these tumor cells corresponds to different differentiation status as in amelogenesis. This is a reason as to why most positive cells in ameloblastoma are round in shape, while negative are columnar cells.

Juuri E et al. [29] showed that SOX2 was expressed in the epithelial cells of follicular and plexiform ameloblastoma diffusely. However, Lei et al. [30] found that SOX2 staining was essentially negative in most ameloblastoma, with occasional positivity in tumor cells. In contrast, it was diffusely and strongly positive in the tumor islands of ameloblastic carcinoma. They assumed that SOX2 might be the potential marker for dysplastic change in ameloblastic neoplasms. In our investigation, when using the antibody used by Lei et al., we found that the islands, anastomosing cords, and cystic lining of the tumor display prominent scattered nuclear stains, especially in the peripheral cells. The numbers of positive cells were not as rare as they pointed out. Most of ameloblastomas arise from jawbones. For this reason, variable amounts of bone fragments are included in specimens obtained from surgical procedures, and these specimens must be subjected to decalcification for tissue processing. In our experience, the acid affected the consequence of immunohistochemistry largely, resulting in false negative staining. This might be the reason for why the cases of ameloblastoma were mostly negative to SOX2 immunostaining in the study published by Lei et al. Therefore, we excluded the specimen that had been subjected to decalcification. We only included cases collected from biopsy or enucleation without the decalcified procedure in this study. Since many of collected cases were gathered from a biopsy specimen, the amount of tumor within formalin fixed paraffin embedded sections was limited. The labeling indices counted on sections of biopsy might not really reflect the expression of the whole tumor. We had included several cases from biopsy and further surgery of identical patients for investigation, and we found that there is an existing discrepancy of expression levels in different tissue sampling. Nevertheless, the influence of sampling error was difficult to determinate and avoid.

Ki-67 (MIB-1) is a well-established proliferative marker used in immunohistochemistry. The labeling indices for Ki-67 in follicular, plexiform, and unicystic ameloblastomas were within the range of several studies previously published [36,37,38]. We exhibited that the Ki-67+ and SOX2+ cells belonged to a different population of tumor cells, and SOX2+ cells were almost negative for Ki-67. Double-labeling cells in immunofluorescence were noted in one case with soft tissue invasion and two cases with inflammatory condition. In these three cases, the labeling indices of SOX2 were extremely high (Figure 4(D1,E1)), which resembled the expression pattern of squamous epithelium (Figure 4F1). Transformation of tumor morphology was seen in two plexiform cases with inflammatory cells infiltrate, the appearance of the tumor strands was identical to proliferative squamous epithelium. We proposed that the tumor behavior and inflammatory condition might affect the amount of SOX2 expressing cells within a tumor.

Most of the tumor cells were exclusively positive for SOX2 or Ki-67 in immunohistochemistry and immunofluorescence. This suggested that these SOX2+ cells might be quiescent cells. Though ameloblastoma is a benign tumor, it is notorious for a high recurrent rate and locally invasive behavior. Vanner et al. displayed the SOX2+ cells were quiescent compared with other rapid-cycling tumor cells, and these SOX2+ cells drove tumor regrowth after treatment in medulloblastoma [39]. It is possible that these quiescent SOX2+ tumor cells give rise to the recurrent lesion in ameloblastoma. Several surveys indicate the surgical methods affect the prognosis of ameloblastoma largely [1,38]. In our collected cases, 13 cases were recurrent tumors; nine of them had well documented surgical methods. Seven cases were treated by enucleation, and two underwent bone tumor excision in the previous surgery. There was no recurrence in cases treated by bloc resection. Some tumor nests might be remained when the tumor was not adequately removed. SOX2 expressing cells remaining in these nests might give rise to the recurrent disease. On the other hand, SOX2 was thought to be a prognostic marker for patients with breast [22], colorectal [20,26], gastric cancer [24], and melanoma [40]. More than one third of sinonasal carcinoma harbored SOX2 amplification, and these cases were more likely to relapse after primary therapy [25]. In our study, when comparing the SOX2 labeling indices between non-recurrent and the recurrent group, or cases with further recurrent lesion and cases without recurrence, no significant difference was noted. However, when comparing the paired primary and recurrent lesions of same patient, we found that the labeling indices were much higher in recurrent tumor than the primary one (Figure 5). This finding reflects the increment of SOX2+ cells after treatment in medulloblastoma by lineage tracing [39], and implies that SOX2+ population is responsible for the relapse of ameloblastomas.

In a transplantation study of skin squamous cell carcinoma (SCC) in mice, they showed that SOX2 marked skin SCC tumor-propagating cells. They also found SOX2 was essential for skin tumor maintenance [14]. In oral SCC, Chou et al. [41] revealed the downregulation of SOX2 led to reduced proliferation, self-renewal, and tumorigenicity in oral cancer stem cells (CSCs). By targeting SOX2, the tumourigenicity and EMT traits were decreased in oral CSCs [41], and growth of medulloblastoma was also inhibited [39]. In our experiment, the cell viability was reduced in both ameloblastoma cell lines by silencing SOX2. Collectively, the role of SOX2-positive cells in ameloblastoma may be tumor-propagating and a driver of recurrence. Considering the high recurrent rate and locally invasive nature of ameloblastoma, patients who have recurrence will encounter extended surgical treatment and reconstruction. Recently, peptide aptamer targeting SOX2 displayed inhibition of proliferation and migration of esophageal SCC [42]. Since SOX2 exists in numerous tissues, local delivery of drug targeting SOX2 may be a potential therapeutic option for recurrent or unresectable ameloblastic neoplasms with amplification of SOX2.

BRAF inhibitor with or without MEK inhibitor for treatment of unresectable or metastatic melanoma and metastatic non-small cell lung cancer with BRAF(V600E) mutation were approved by the U.S. Food and Drug Administration [43]. A large proportion of ameloblastomas harbor BRAF(V600E) mutation, the BRAF inhibitor with or without the combination of MEK inhibitor was applied in several cases of unresectable or recurrent ameloblastoma harboring BRAF(V600E) mutation [44,45,46,47]. In these case series, this targeted therapy to BRAF mutated ameloblastomas revealed a significant response. The therapy through BRAF/MEK inhibition might serve as a neoadjuvant and/or adjuvant therapeutic option in unresectable ameloblastic neoplasms with BRAF(V600E) mutation. However, after long-term application of BRAF inhibitor, residual tumor unresponsive to treatment has been reported [45,46,47]. Relapse was also frequently present in several cancers under treatment with BRAF inhibitor [48,49]. In our investigation, we found the drug resistant clone of ameloblastoma cells revealed upregulation of SOX2. The amplification of SOX2 might assist the maintenance of stemness property and spur further recurrence.

In this study, we sorted out the expression patterns of SOX2+ and Ki-67+ cells in dental follicle and ameloblastoma, confirmed a high rate of BRAF(V600E) mutation of ameloblastomas in Asian patients, and these mutated cases showed significantly more SOX2-positive cells. Several findings indicated that the SOX2 expressing cells played significant roles in propagation and recurrence of ameloblastoma and suggested BRAF(V600E) mutation may contribute to the expansion of SOX2-positive cell compartment. However, the role of SOX2 in clinical behavior of ameloblastoma and the cross-talks between SOX2 and MAPK pathway have not been clarified. It needs further investigation to uncover what roles SOX2 and BRAF play in the tumor biology of ameloblastoma. Since ameloblastoma is a benign tumor, the establishment of an animal model and primary tumor cell culture for ameloblastoma are difficult to execute. It may take time to overcome these problems for further experiments.

## Figures and Tables

**Table 1:** Clinical parameters in three histologic patterns of ameloblastoma.

	Follicular Type (*n* = 27)	Plexiform Type (*n* = 22)	Unicystic Type (*n* = 25)
Age (year)			
<20	1	8	9
21 to 40	9	8	12
≥41	17	6	4
Gender			
Men	15	18	12
Women	12	4	13
Location			
Maxilla	5	0	0
Mandible	22	22	25
Bone perforation			
Present	16	14	13
Absent	2	6	4
Root resorption			
Present	15	13	11
Absent	7	7	11
Size of lesion ^a^			
<100,000	7	9	15
100,001 to 200,000	10	5	8
≥200,001	5	6	1

^a^ The numbers in the row of lesion size were obtained from measuring pre-surgical panoramic X-ray with ROI manager of Image J (National Institutes of Health, Bethesda, MD, USA).

**Table 2:** Expression status of SOX2 in remnants of odontogenic epithelial rests.

Case Number	Labeling Indices of SOX2 Immunostain (%)
DF-1	33.8
DF-2	47.3
DF-3	45.7
DF-4	47.8
DF-5	59.2
DF-6	34.1
	Mean ± S.D. (%): 44.7 ± 9.6 Median (%): 46.5

**Table 3:** Immunohistochemistry in three histologic patterns of ameloblastoma.

	Follicular Type (*n* = 27)	Plexiform Type (*n* = 22)	Unicystic Type (*n* = 25)
Ratio of high columnar cells in SOX2+ cells			
<2	20	21	20
≥2	7	1	5
Labeling index of SOX2 (%)			
<10	17	5	8
11 to 20	3	4	7
>20	7	13	10
Labeling index of Ki-67 (%)			
<3	21	11	14
3 to 6	3	6	7
>6	3	5	4

**Table 4:** Comparison of expression status of SOX2 in three types of ameloblastomas.

Histologic Type	Mean ± S.D. (%)	Median (%)	Comparing with	*p* ^a^
Follicular type	17.2 ± 21.9	6.2	Plexiform type	0.031
			Unicystic type	0.107
Plexiform type	28.8 ± 22.1	23.3	Follicular type	0.031
			Unicystic type	0.216
Unicystic type	20.3 ± 16.5	14.2	Follicular type	0.107
			Plexiform type	0.216

^a^ Mann–Whitney U test.

**Table 5:** Comparison of expression status of Ki-67 in three types of ameloblastomas.

Histologic Type	Mean ± S.D. (%)	Median (%)	Comparing with	*p* ^a^
Follicular type	2.5 ± 2.0	2.0	Plexiform type	0.048
			Unicystic type	0.674
Plexiform type	4.1 ± 3.3	3.0	Follicular type	0.048
			Unicystic type	0.179
Unicystic type	3.3 ± 3.1	1.8	Follicular type	0.674
			Plexiform type	0.179

^a^ Mann–Whitney U test.

**Table 6:** SOX2-positive rate distribution among BRAF(V600E) wild type and mutant in different subtypes of the ameloblastoma cases in this study.

Ameloblastoma	BRAF(V600E) Wild Type			BRAF(V600E) Mutant		
Subtype	Follicular	Plexiform (*n* = 2)	Unicystic (*n* = 5)	Follicular (*n* = 18)	Plexiform (*n* = 14)	Unicystic (*n* = 16)
SOX2-positive cells	-	7.25% ± 0.03%	6.3% ± 0.02%	21.74% ± 0.22%	35.05% ± 0.2%	18.87% ± 0.15%
Average	6.57% ± 0.02%			24.54% ± 0.21%		

## Supplementary Materials

The following supporting information can be downloaded at: https://www.mdpi.com/article/10.3390/jpm12010077/s1, Table S1: Primers used in targeted cDNA sequencing and real-time RT–PCR, Table S2: Correlation between the expression statuses of SOX2 and Ki-67, Table S3: Correlation of expression status of SOX2 and Ki-67 with clinical parameters, Table S4: Correlation of size of lesion to root resorption and bone perforation in three types of ameloblastoma, Table S5: Comparison of expression status of SOX2 and Ki-67 in recurrent lesions, Table S6: Comparison of expression status of SOX2 and Ki-67 in cases with further recurrent lesions, Table S7: Comparison of expression status of SOX2 and Ki-67 in mural type and luminal/intra-luminal type unicystic ameloblastomas, Table S8: Comparison of expression status of SOX2 and Ki-67 in intra-luminal/luminal type and other types of ameloblastomas, Figure S1: Knockdown efficiency of SOX2 in ameloblastoma cell lines, Figure S2: Inhibition of BRAF reduces cell viability of AM1 cells.
