# Supplementary material for: Enrichment of SOX2-Positive Cells in BRAF V600E Mutated and Recurrent Ameloblastoma
Source: J Pers Med. 2022 Jan 8;12(1):77. doi: 10.3390/jpm12010077 (PMC8780877; doi:10.3390/jpm12010077)
Supplement: Supplementary file 1 [file jpm-12-00077-s001.zip › jpm-1511076-supplementary.pdf]

Supplementary Materials

Enrichment of SOX2-Positive Cells in Ameloblastoma Patients with Tumor Recurrence and BRAF V600E Mutated Cases

Chih-Huang Tseng [1,2,3], Pei Hsuan Lu [4], Yi-Ping Wang [3,4,5]
and Julia Yu Fong Chang [3,4,5,*]

1 Division of Oral Pathology & Maxillofacial Radiology, Department of Dentistry, Kaohsiung Medical University Hospital, Kaohsiung, Taiwan; 1050565@kmuh.org.tw
2 Oral & Maxillofacial Imaging Center, College of Dental Medicine, Kaohsiung Medical University, Kaohsiung, Taiwan
3 Graduate Institute of Clinical Dentistry, School of Dentistry, National Taiwan University, Taipei, Taiwan; ypwang0530@ntu.edu.tw
4 Department of Dentistry, National Taiwan University Hospital, College of Medicine, National Taiwan University, Taipei, Taiwan; R04422025@ntu.edu.tw
5 Graduate Institute of Oral Biology, School of Dentistry, National Taiwan University, Taipei, Taiwan; jyfchang@ntu.edu.tw
* Correspondence: jyfchang@ntu.edu.tw

Table S1. Primers used in targeted DNA sequencing and real-time RT–PCR

DNA sequencing

Primer name	Sequences (5' to 3')
BRAF-ex15F	TGCTTGCTCTGATAGGAAAATG
BRAF-ex15R	AGCATCTCAGGGCCAAAAAT

Real-time RT–PCR

Primer name	Sequences (5' to 3')
hBRAF(V600E)-F	ATTGCACGACAGACTGCAC
hBRAF(V600E)-R	CCCACTCCATCGAGATTTCT
hSOX2-F	GCTACAGCATGATGCAGGACCA
hSOX2-R	GCGAGCTGGTCATGGAGTT
GAPDH-F	GCACCGTCA AGGCTG AGA AC
GAPDH-R	TGGTGGTGA AGACGCCAGT

Table S2. Correlation between the expression statuses of SOX2 and Ki-67.

Histologic type	*p* [a]
Follicular type	0.324
Plexiform type	0.501
Unicystic type	0.103

[a] Spearman's correlation coefficient.

Table S3. Correlation of expression status of SOX2 and Ki-67 with clinical parameters[a].

Histologic Type	Marker Expressed	Clinical parameters		
		**Size of lesion**	**Root resorption**	**Bone perforation**
Follicular type	SOX2	p=0.336	p=0.731	p=0.837
	Ki-67	p=0.490	p=0.298	p=0.261
Plexiform type	SOX2	p=0.207	p=0.663	p=0.968
	Ki-67	p=0.652	p=0.843	p=0.904
Unicystic type	SOX2	p=0.840	p=0.483	p=0.245
	Ki-67	p=0.223	p=0.151	p=0.785

[a] Spearman's correlation coefficient.

Table S4. Correlation of size of lesion to root resorption and bone perforation in three types of ameloblastoma[a].

**Histologic type**		**Root resorption**	**Bone perforation**
Follicular type	Size of lesion	*p*=0.891	*p*=0.441
		Root resorption	Bone perforation
Plexiform type	Size of lesion	*p*=0.636	*p*=0.053
		Root resorption	Bone perforation
Unicystic type	Size of lesion	*p*=0.562	*p*=0.703

[a] Spearman's correlation coefficient.

Table S5. Comparison of expression status of SOX2 and Ki-67 in recurrent lesions[a].

**Histologic type**	**Case number**		**Labeling indices of SOX2 (%)**	**Labeling indices of Ki-67 (%)**	**Comparing SOX2 expression status with other cases without recurrent event in the same type**	**Comparing Ki-67 expression status with other cases without recurrent event in the same type**
**Follicular type**	AB-3		42.7	1.3		
	AB-6		38.4	1.2		
	AB-7		2.8	1.7		
	AB-8		4	1.0		
	AB-12		4	0.6		
	AB-14		6.2	2.0		
	AB-16		2.2	7.2		
	AB-25		58.2	5.4		
	AB-26		7.2	3.0		
		Mean ± S.D. (%)	18.4 ± 21.7	2.6 ± 2.3	*p*=0.958	*p*=0.916
**Plexiform type**	AB-31		27.4	2.6		
	AB-33		62.7	8.0		
	AB-37		41.7	2.6		
		Mean ±	43.9 ± 17.8	4.4 ± 3.1	*p*=0.258	*p*=0.765

		S.D. (%)				
**Unicystic type**	AB-59		8.5	5.5	-	-

[a] Mann-Whitney U test.

Table S6. Comparison of expression status of SOX2 and Ki-67 in cases with further recurrent lesions[a].

**Histologic type**	**Case number**		**Labeling indices of SOX2 (%)**	**Labeling indices of Ki-67 (%)**	**Comparing SOX2 expression status with other cases without recurrent event in the same type**	**Comparing Ki-67 expression status with other cases without recurrent event in the same type**
**Follicular type**	AB-16		2.2	7.2		
	AB-19		26.5	2.2		
	AB-25		58.2	5.4		
		Mean ± S.D. (%)	29.0 ± 28.1	5.8 ± 3.9	p=0.546	p=0.146
**Plexiform type**	AB-31		27.4	2.6		
	AB-37		41.7	2.6		
	AB-39		15.0	2.9		
	AB-43		9.1	1.9		
		Mean ± S.D. (%)	23.3 ± 14.4	2.5 ± 0.4	p=0.763	p=0.635

[a] Mann-Whitney U test.

Table S7. Comparison of expression status of SOX2 and Ki-67 in mural type and luminal/intra-luminal type unicystic ameloblastomas[a].

Subtypes of unicystic ameloblastoma	SOX2 expression status	Ki-67 expression status
Mural type	p=0.452	p=0.971
Intra-luminal/luminal type		

[a] Mann-Whitney U test.

Table S8. Comparison of expression status of SOX2 and Ki-67 in intra-luminal/luminal type and other types of ameloblastomas[a].

Histologic type	Sox2 expression status Mean ± S.D. (%)	p	Ki-67 expression status Mean ± S.D. (%)	p
Intra-luminal/luminal type	13.5 ± 9.5	0.653	2.8 ± 2.3	0.809
Other types	22.2 ± 21.0		3.2 ± 2.7	

[a] Mann-Whitney U test.

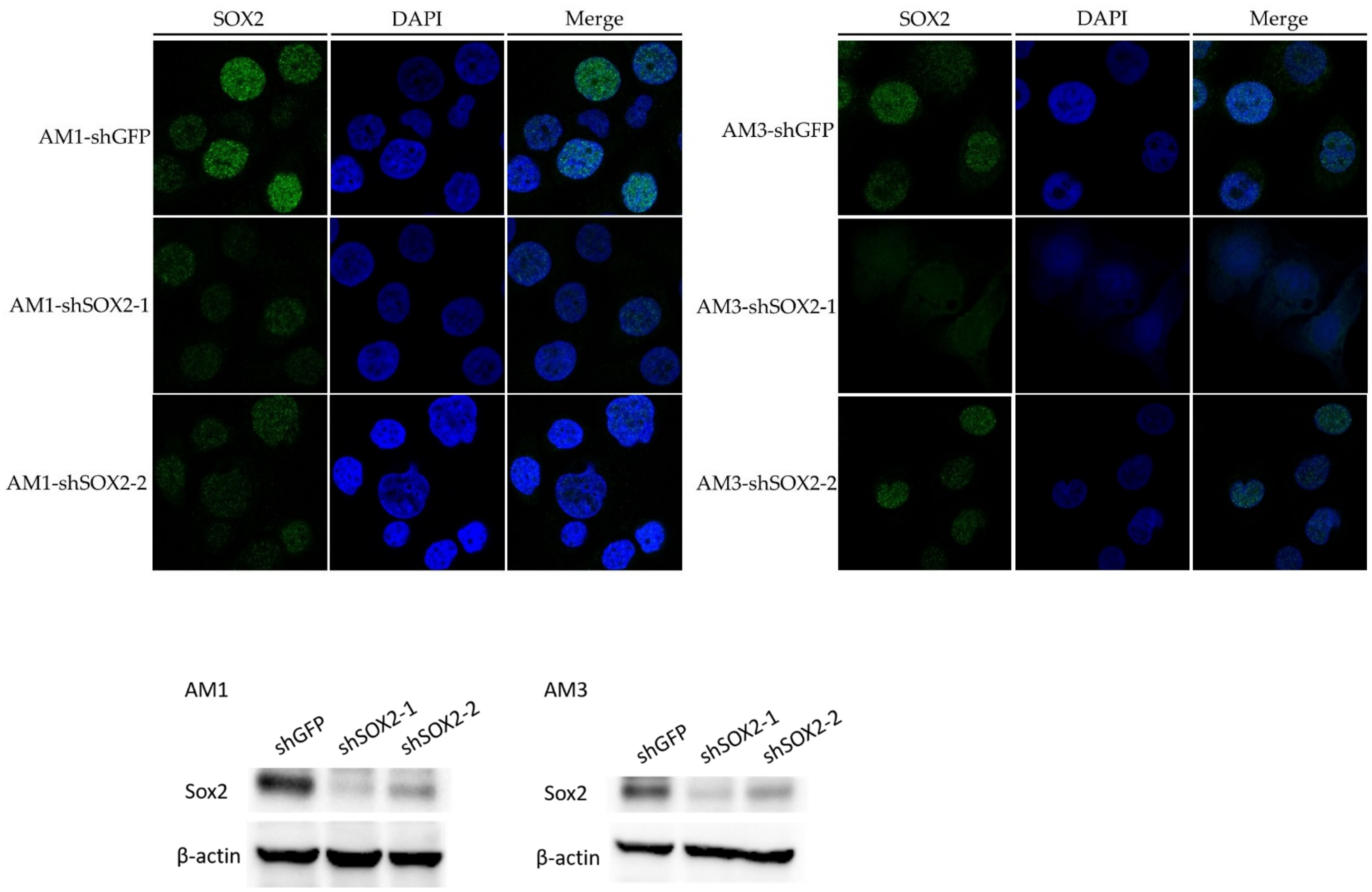

Figure S1. Knockdown efficiency of SOX2 in ameloblastoma cell lines.

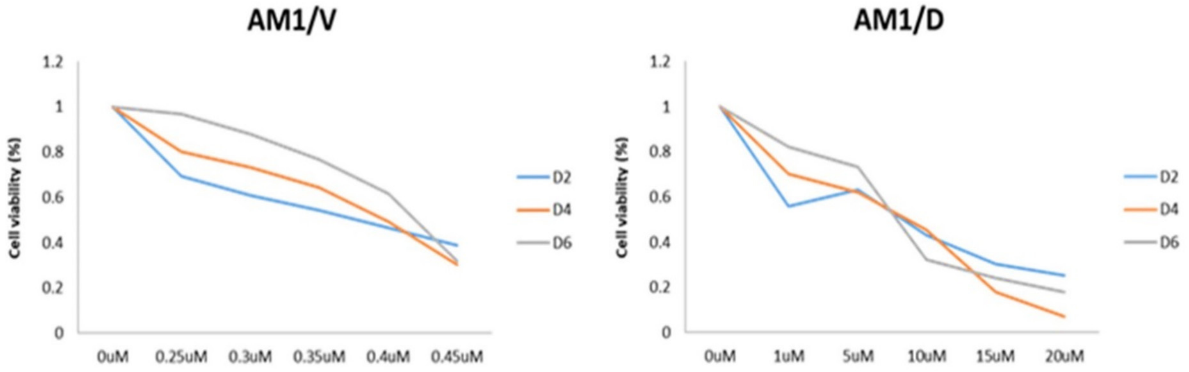

Figure S2. Inhibition of BRAF reduces cell viability of AM1 cells. AM1 cell was treated with BRAF inhibitors (**V:** vemurafenib; **D**: dabrafenib) for 2, 4 and 6 days then analysis the cell viability.
